# A Case Report of Severe Thrombocytopenic Purpura during Neoadjuvant Pembrolizumab Administration for Triple-Negative Breast Cancer

**DOI:** 10.70352/scrj.cr.24-0036

**Published:** 2025-02-01

**Authors:** Ryoko Semba, Shiori Tohyama, Yumiko Ushiyama, Fumi Murakami, Sakiko Harada, Kanako Ogura, Junichiro Watanabe

**Affiliations:** 1 Department of Breast Oncology, Juntendo University Faculty of Medicine, Tokyo, Japan; 2 Department of Hematology, Juntendo University Faculty of Medicine, Tokyo, Japan; 3 Department of Human Pathology, Juntendo University Faculty of Medicine, Tokyo, Japan

**Keywords:** hematologic irAEs, pembrolizumab, immune thrombocytopenic purpura

## Abstract

**INTRODUCTION:**

Immune thrombocytopenic purpura (ITP) is one of the immune-related adverse events (irAEs) related to immune checkpoint inhibitors (ICIs). Here, we report a case of a 51-year-old woman with triple-negative breast cancer (TNBC) who experienced severe thrombocytopenic purpura during the neoadjuvant chemotherapy (NAC), including pembrolizumab.

**CASE PRESENTATION:**

A 51-year-old woman was diagnosed with Stage II B TNBC and underwent NAC using pembrolizumab + paclitaxel + carboplatin. Her blood test on cycle 4, day 15 (C4D15) showed a significant decrease in platelets to <2000/μL accompanied by overt bleeding tendency. She was hospitalized for further investigation and treatment. Her platelet count recovered after platelet concentrate transfusion and corticosteroid administration. Her bone marrow examination showed normal cellularity, and she was judged as ITP. Due to the event and good clinical response to NAC, she underwent a right partial mastectomy and axillary lymph node dissection without completion of the planned NAC. The surgical specimen showed a complete pathological response.

**CONCLUSIONS:**

Thrombocytopenia is known as one of the hematologic irAEs; however, severe thrombocytopenia with a bleeding tendency is rarely reported. Sufficient explanations to patients and appropriate referral to other related departments are important for earlier detection and treatment of irAE.

## Abbreviations

HE
: hematoxylin and eosin
ICIs
: immune checkpoint inhibitors
IrAEs
: immune-related adverse events
ITP
: immune thrombocytopenic purpura
NAC
: neoadjuvant chemotherapy
pCR
: pathological complete response
PSL
: prednisolone
TNBC
: triple-negative breast cancer

## INTRODUCTION

Immune checkpoint inhibitors (ICIs) have been widely used to manage various malignancies. However, according to those modes of action, that is, suppression of immune-suppressor, immune-related adverse events (irAEs) may accompany and cannot be predictable.^1)^ Compared to common irAEs, such as endocrinopathy and interstitial lung diseases,^2)^ immune-related hematologic disorders have been rarely reported. Unlike conventional cytotoxic agents, hematologic irAEs require immunosuppressive treatments using corticosteroids in addition to suspending ICI, which will be life-threatening.^3)^ Because of the rarity of hematological irAEs, they have not been fully analyzed, and many parts of them remain unexplained.

## CASE PRESENTATION

**Fig. 1:**
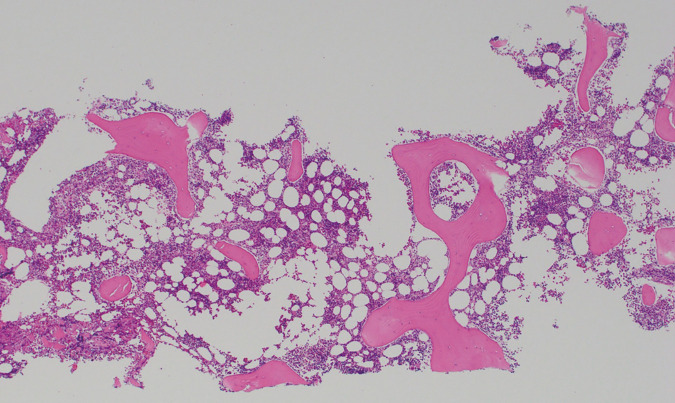
Hematoxylin and eosin staining of the bone marrow biopsy. There were no atypical cells in the bone marrow biopsy.

**Fig. 2:**
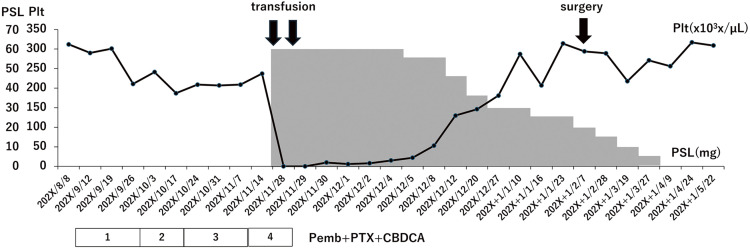
Treatments after thrombocytopenic purpura and changes in platelet count. The platelet count recovered over time after starting to give platelet concentrate and prednisolone.

A 51-year-old woman with a right breast mass went to her family doctor, and she was diagnosed with triple-negative, invasive ductal carcinoma. She was referred to our hospital for further examination and treatment. Her family history included her mother in her 70s with breast cancer, a medical history of asymptomatic ventricular septal defect, hypertension, and diabetes. Ultrasonography scans and contrast-enhanced breast MRI scans revealed a 45 mm-sized mass in her right breast and three enlarged lymph nodes in her right axilla. The results of fine-needle aspiration cytology of the axillary lymph nodes indicated findings suggestive of metastatic adenocarcinoma. Since she was diagnosed with T2N1M0, stage IIB triple-negative breast cancer, she underwent neoadjuvant chemotherapy (NAC) according to a KEYNOTE-522 study using pembrolizumab.^4)^ She received 200 mg/body of pembrolizumab on day 1, 80 mg/m^2^ on days 1, 8, 22, and 1.5 are area under the curves (AUCs) of carboplatin on days 1, 8, and 15 repeated by 3 weeks. On cycle 4, day 1, she experienced epistaxis and bloody stool. Due to a significant decrease in platelet count to <2000/μL and overt bleeding tendency, she was hospitalized for further examination and treatment. Her hematological data showed no abnormalities in blood counts other than platelets consistently from the initiation of chemotherapy. The more detailed blood tests ruled out systemic diseases accompanying thrombocytopenia, such as systemic lupus erythematosus or antiphospholipid syndrome. Bone marrow aspiration was performed by a hematologist. The results of the hematoxylin and eosin (H&E) staining of the bone marrow biopsy (**Fig. 1**) showed normal cellularity and no fibrosis. The results, including the result of the bone marrow smear, did not positively support the diagnosis of typical immune thrombocytopenic purpura (ITP) because of no increase in megakaryocytes nor loss of platelet adhesion; there was no positive evidence of malignant cells or abnormal blood cells, which supports bone marrow carcinomatosis or myelodysplastic syndrome. Splenomegaly and hepatic cirrhosis were ruled out from the CT scan. Finally, we judged the events as pembrolizumab-related ITP. Ten units of platelet concentrate were transfused and 60 mg/day of prednisolone (PSL) was started. The platelet count recovered over time, and the dose of PSL was gradually tapered. The patient was discharged on the 16th of the hospital day (**Fig. 2**). Regarding her clinical course, NAC was discontinued, and she underwent a right partial mastectomy and axillary lymph node dissection when PSL was tapered to 25 mg/day. No excessive bleeding was noted during her mastectomy. She achieved pathological complete response (pCR) as ypT0N0. PSL was constantly tapered by 5 mg every 2 weeks and permanently discontinued 4 months after the initiation; however, according to the patient’s request, adjuvant pembrolizumab was omitted, and she underwent postmastectomy radiation therapy.

## DISCUSSION

Although not reported for pembrolizumab, a report on hematologic irAEs with ICIs such as nivolumab and ipilimumab accounted for 3.6% of any grades and 0.7% of grade ≥3 of all irAEs, and ITP was most common (29% of grade ≥2 of hematologic irAEs) followed by pancytopenia and aplastic anemia.^5)^ A report focusing on ICI-related thrombocytopenia showed that 8.6% of all patients treated with ICI developed grade ≥3 thrombocytopenia, and grade ≥3 immune-related thrombocytopenia could lead to worse overall survival due to the increased risk of clinically significant bleeding events.^6)^

While corticosteroids are commonly introduced to manage thrombocytopenia, 20% of patients were refractory to corticosteroid therapy.^7)^ If the platelet count responds promptly, corticosteroids can be tapered gradually following a standard course of 2–4 weeks. In cases where first-line treatment is ineffective, options such as IVIg, rituximab, splenectomy, thrombopoietin receptor agonists, or second-line immunosuppressive agents like cyclosporine or azathioprine may be considered.^8,9)^ In this case, we consulted with hematologists and started high-dose PSL (1 mg/kg) immediately after the suspect of hematologic irAE. It is sometimes difficult to distinguish ITP from other diseases that may accompany severe thrombocytopenia because an increase in megakaryocytes is not always prominent in ITP patients. In addition to the differential diagnoses already mentioned in the Case Presentation section, the possibility of myelosuppression associated with chemotherapy was quite unlikely because of the proper administration of chemotherapy and clinical findings.

The patient had been aware of epistaxis about 1 week before the day of her scheduled visit. If the collaborative support between healthcare providers and the patient had been established, the irAE may have been discovered earlier. Since irAE may cause various symptoms during, or even after, the ICI therapy, medical professionals should inform their patients not to hesitate to consult her/his doctor immediately when she/he feels unusual symptoms such as frequent epistaxis, irregular genital bleeding, or melena.

**Fig. 3:**
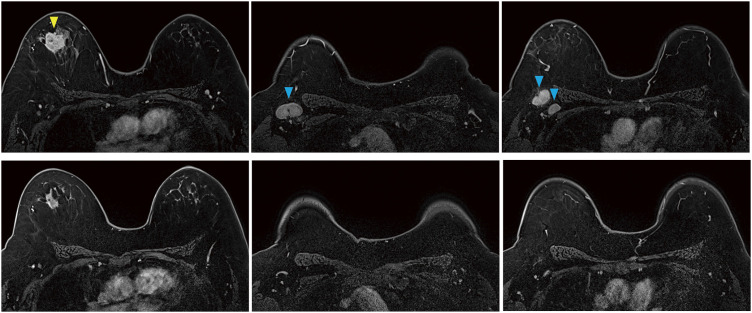
Breast MRI images before (upper) and after (lower) NAC (fat suppression T1-weighted early phase). The tumor and lymph nodes with strong enhancements (yellow and blue arrowheads) disappeared after NAC. NAC, neoadjuvant chemotherapy

Although the remaining pembrolizumab + epirubicin + cyclophosphamide chemotherapy scheduled for preoperative chemotherapy could not be administered, the chemotherapy showed sufficient effectiveness as radiological CR at the preoperative workup, consistent with pCR (**Fig. 3**) which supported the patient’s judgment avoiding adjuvant pembrolizumab; however, there is a discussion regarding the omission of adjuvant pembrolizumab in a patient who achieved pCR.^10)^ There have been reports that the same irAEs occurred in approximately one-quarter of all cases after the rechallenge of the same ICI after irAEs.^11)^ Therefore, it is mandatory to make careful judgments regarding re-administration of ICI on a case-by-case basis. In this case, we considered that adjuvant pembrolizumab may increase the risk of recurrent thrombocytopenia, and it is highly likely to exceed the patient’s benefit.

## CONCLUSION

Although severe ITP, which accompanied hemorrhagic events, occurred during neoadjuvant ICI administration against an early-stage TNBC patient, a definite diagnosis was made promptly after consulting with a hematologist, and the symptoms were improved by corticosteroid administration and an appropriate platelet transfusion. When clinical symptoms or laboratory findings show the possibility of irAEs with ICIs, it is important to cooperate with doctors from other related departments immediately and provide appropriate definite diagnosis and efficient treatment.

## DECLARATIONS

### Funding

Not applicable.

### Authors’ contributions

RS, ST, YU, FM, and SH treated these patients and provided clinical information.

KO conducted the histological assessments.

RS wrote the manuscript.

JW reviewed and edited the manuscript.

All authors contributed to discussions, agreed on the final version of the manuscript for submission, and agreed to be responsible for this study.

### Availability of data and materials

Not applicable.

### Ethics approval and consent to participate

Not applicable.

### Consent for publication

Written informed consent was obtained from the patients for publication of this case report.

### Disclosure statement

Junichiro Watanabe receives personal fees from MSD for activities outside the scope of the submitted work. The other authors declare that there are no competing interests regarding the publication.
